# Retrospective Clinical and Radiological Comparison of Intradiscal Ozone and Ozone + PRP Therapy Results in Patients with Intervertebral Disc Degeneration

**DOI:** 10.4274/TJAR.2025.241831

**Published:** 2025-10-14

**Authors:** Gülseli Berivan Sezen, Osman Boyalı, Burak Karip, Selman Aktaş, Eyüp Can Savrunlu, Mourat Chasan, Necati Kaplan, Erdinç Civelek, Serdar Kabataş

**Affiliations:** 1 Giresun Training and Research Hospital, Clinic of Neurosurgery, Giresun, Türkiye; 2 University of Health Sciences Türkiye, Gaziosmanpaşa Training and Research Hospital, Clinic of Neurosurgery, İstanbul, Türkiye; 3 University of Health Sciences Türkiye, Hamidiye Faculty of Medicine, Department of Anatomy, İstanbul, Türkiye; 4 University of Health Sciences Türkiye, Hamidiye Faculty of Medicine, Department of Biostatistics, İstanbul, Türkiye; 5 Optimed International Çorlu Hospital, Clinic of Neurosurgery, Tekirdağ, Türkiye

**Keywords:** Intervertebral disc degeneration, intradiscal, low back pain, ozone, platelet-rich plasma

## Abstract

**Objective:**

This retrospective study aimed to evaluate and compare the clinical efficacy of intradiscal ozone therapy (OT) against a combination therapy of ozone and platelet-rich plasma (PRP) in patients diagnosed with intervertebral disc degeneration (IVDD).

**Methods:**

The study included a cohort of 50 patients, divided equally into two groups of 25, who received either intradiscal OT or ozone + PRP combination therapy between February 2022 and February 2023. The sample comprised 20 females and 30 males, with ages ranging from 19 to 76 years (mean age 48.8). Pain intensity was measured using the visual analog scale (VAS), while disability levels were assessed using the oswestry disability index (ODI) prior to treatment and at 1, 3, and 6 months post-treatment. Additionally, lumbar magnetic resonance imaging was conducted at the 3-month mark post-treatment, with evaluations based on the Pfirrmann disc degeneration classification.

**Results:**

Significant improvement in both VAS and ODI scores was observed in both treatment groups (*P* < 0.001). The ozone + PRP combination therapy group exhibited no statistically significant difference in VAS and ODI scores compared to the ozone-only group (*P* > 0.05).

**Conclusion:**

Intradiscal OT and the ozone + PRP combination therapy represent effective minimally invasive treatment options for patients suffering from IVDD, yielding substantial clinical benefits with minimal side effects. That is why it is suggested as a potential preferred therapeutic approach prior to the consideration of surgical interventions.

**Main Points**

• The study compared the effectiveness of intradiscal ozone and ozone + platelet-rich plasma (PRP) combination treatments for intervertebral disc degeneration (IVDD).

• Ozone therapy (OT) and ozone + PRP treatments both significantly improved pain and disability stages in patients with IVDD.

• There is no statistically significant difference between OT and ozone + PRP therapies in pain and disability reduction, indicating that both are equally effective.

## Introduction 

Low back pain (LBP) is a widespread condition, impacting up to 85% of individuals at some stage in their lives.^1^ While the majority of cases resolve spontaneously within a few weeks, a notable percentage of patients progress to develop chronic symptoms that can significantly diminish their quality of life.^2^ Intervertebral disc degeneration (IVDD) is a prevalent etiology of chronic LBP, characterized by morphological and biochemical alterations in the disc tissue, leading to impaired mechanical function and pain. The management of IVDD presents considerable challenges due to the limited self-healing capacity of the disc, primarily attributed to its inadequate vascularization and the hypoxic microenvironment present within the disc.^3^ As insights into the pathophysiology of IVDD continue to evolve, minimally invasive interventions such as intradiscal ozone therapy (OT) and platelet-rich plasma (PRP) therapy have garnered interest for their potential to alleviate pain and restore functionality.^4, 5^ It has been reported that PRP induces the release of bioactive proteins that influence macrophages, mesenchymal stem cells, osteoblasts and/or annulus fibrosus cells, as well as nucleus pulposus cells accelerating the absorption of necrotic tissues and promoting regeneration.^6^ OT, which entails the injection of a gas mixture of oxygen and ozone into the affected disc, is thought to exert its therapeutic effects through the induction of oxidative stress, resulting in a reduction of disc volume and alleviation of nerve compression.^7, 8^ Conversely, PRP therapy employs autologous growth factors to facilitate tissue repair and mitigate inflammation.^9, 10^ Recent investigations indicate that the synergistic application of these therapies may enhance their individual therapeutic effects, thereby providing a more effective treatment alternative for patients suffering from IVDD.^11^ This study aims to retrospectively compare the clinical and radiological outcomes of intradiscal OT with those of the ozone and PRP combination therapy in patients diagnosed with IVDD.

## Methods

Ethical approval was received from the University of Health Sciences Türkiye, Sultan 2. Abdülhamid Han Training and Research Hospital, GETAT Clinical Research Ethics Committee (approval no.: SBllSAH-GETAT 2023-048, date: 13.12.2023). This retrospective analysis encompassed 50 patients (20 females and 30 males) aged between 19 and 76 years (mean age 48.8), who received either intradiscal OT or ozone + PRP combination therapy from February 2022 to February 2023. Patient selection was based on the presence of LBP that was unresponsive to conservative management, magnetic resonance imaging (MRI) findings indicative of disc protrusion or bulging, Pfirmann grade 3-6 and a visual analog scale (VAS) score of 5 or higher. Exclusion criteria included pregnancy, motor deficits, bleeding diathesis, glucose-6-phosphate dehydrogenase deficiency, active infection, and previous lumbar surgery. Pain intensity was quantified using the VAS, while disability levels were evaluated using the oswestry disability index (ODI) prior to treatment and at 1, 3, and 6 months post-treatment. VAS is a subjective pain assessment method in which patients rate their pain level on a scale from 0 (no pain) to 10 (unbearable pain).^12^ ODI is a standardized 10-item questionnaire used to evaluate lower-back pain and functional disability. Patients subjectively score their daily living activities and the limitations they experience in these activities.^13^ Additionally, lumbar MRI assessments conducted at 3 months post-treatment were analyzed according to the Pfirmann disc degeneration classification.

### Procedure

In the study involving patients undergoing ozone + PRP therapy, a total of 20 cc of blood was collected and mixed with 1 cc of citrate per 9 cc of blood. This mixture was subsequently transferred into two 10 cc yellow tubes (sterile and preservative-free tubes) and centrifuged at 3000 rpm for 5 minutes to prepare the PRP. After centrifugation, 2 mL of PRP was obtained.

The patient was then positioned prone on the operating table, with a bilateral pillow placed under the abdomen for support. The procedures were performed under local anaesthesia. Fluoroscopy equipment was prepared for imaging purposes. The puncture site was sterilized (with povidone iodine), and sterile drapes were applied. The target intervertebral disc level was identified using fluoroscopic imaging, and the entry point was marked accordingly. A local anaesthesia solution containing 0.5% lidocaine was administered at the injection site. Under fluoroscopic guidance, utilizing anteroposterior and lateral and oblique views, a 22-gauge, 20 cm spinal needle was inserted posterolaterally into the nucleus pulposus of the disc suspected of causing pain, approximately 10-12 cm lateral to the midline at an angle of 30-45 degrees. For the OT component, 10 mL of an O_2_-O_3_ mixture (20 μg mL^-1^ O_3_) was injected at each disc level. Procedures were performed only with intradiscal injections at a single level for each patient, while transforaminal injections were omitted. In patients receiving the combined ozone and PRP therapy, 2 mL of PRP, was administered first, followed by the 10 mL O_2_-O_3_ mixture.

Following the procedure, the patient was monitored for approximately 2 hours, before being mobilized and discharged after a 5-day rest period, with no complications reported during mobilization.

### Statistical Analysis 

Data were analyzed using two-way analysis of variance (ANOVA) for repeated measures to evaluate pre- and post-treatment scores both within and between groups. Chi-square tests were employed to assess demographic characteristics. All statistical analyses were conducted using SPSS software (version 22; SPSS Inc., Chicago, IL, USA), with a significance threshold set at* P *< 0.05.

## Results 

### Patient Demographics and Baseline Characteristics

The study sample consisted of 50 patients, including 20 females (40%) and 30 males (60%). The mean age of participants was 48.8 years, with an age range spanning from 19 to 76 years.

No statistically significant differences were observed between the two treatment groups concerning age, gender distribution, or baseline VAS and ODI scores, thereby ensuring comparability (*P *> 0.05) (Table 1).

**Figure 1:**
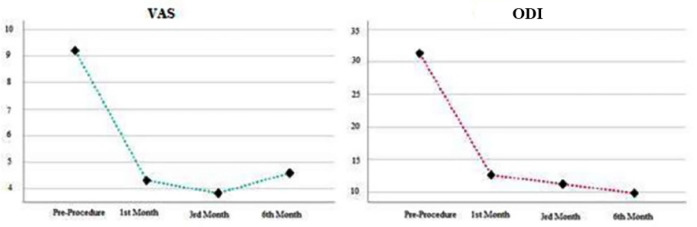
Comparison of VAS and ODI scores in patients receiving ozone therapy. VAS, visual analog scale; ODI, oswestry disability index.

Of the patients receiving OT, 7 were female (28%) and 18 were male (72%). The analysis revealed a statistically significant difference in VAS and ODI scores for patients undergoing isolated OT (*P *< 0.05). Specifically, VAS scores indicated that pre-procedure values were significantly higher than those recorded at the 1^st^, 3^rd^, and 6^th^ months post-procedure (*P *< 0.05) (Table 2). Notably, the lowest VAS score was observed at the 3^rd^ month post-procedure (*P *< 0.05) (Figure 1). ODI scores demonstrated that pre-procedure values were significantly higher than those at the 1^st^, 3^rd^, and 6^th^ months post-procedure (*P *< 0.05), with the lowest ODI score recorded at the 6^th^ month post-procedure (*P *< 0.05) (Figure 1).

**Figure 2:**
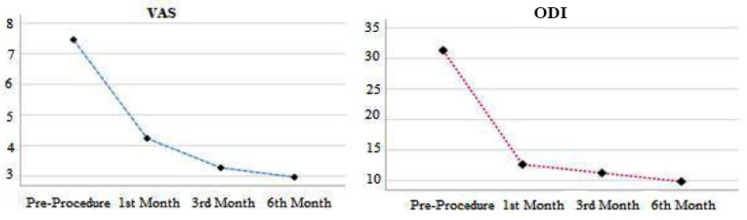
Comparison of VAS and ODI scores in patients receiving ozone + PRP combine therapy. VAS, visual analog scale; ODI, oswestry disability index; PRP, platelet-rich plasma.

Of the patients receiving ozone + PRP treatment, 15 were female (60%) and 10 were male (40%). In patients receiving ozone + PRP treatment, the analysis revealed a statistically significant difference in VAS and ODI scores (*P *< 0.05) (Table 3). The pre-procedure VAS scores were found to be higher than the scores recorded at the 1^st^, 3^rd^, and 6^th^ months post-the procedure (*P *< 0.05). When the post-procedure VAS scores were examined, it was observed that the 6^th^-month VAS score was the lowest (*P *< 0.05) (Figure 2). In ODI scores, it was observed that the pre-procedure scores were higher than the 1^st^, 3^rd^ and 6^th^ month scores after the procedure (*P *< 0.05). The examinations conducted in the 1^st^, 3^rd^, and 6^th^ months post-procedure revealed that the 6^th^ month ODI score was the lowest (*P *< 0.05) (Figure 2).

The VAS and ODI scores at the 1^st^, 3^rd^, and 6^th^ months post-procedure for both treatment groups were statistically significantly lower (*P *< 0.05) compared to the pre-operative scores (Figures 1, 2). However, no statistically significant differences were observed in VAS and ODI scores when comparing the 1^st^, 3^rd^, and 6^th^ months scores within both groups (*P *> 0.05).

### Radiological Outcomes

### Disc Size and Morphology

**Figure 3:**
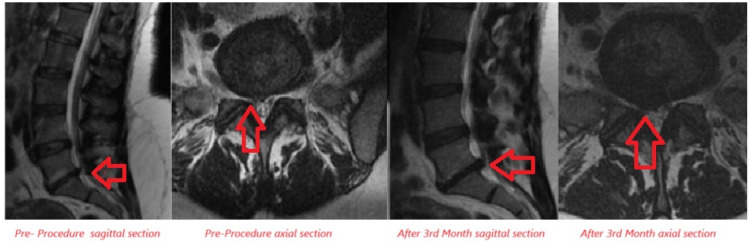
Examination of disc size increase after ozone therapy in lumbar MRI sagittal and axial sections. MRI, magnetic resonance imaging.

**Figure 4:**
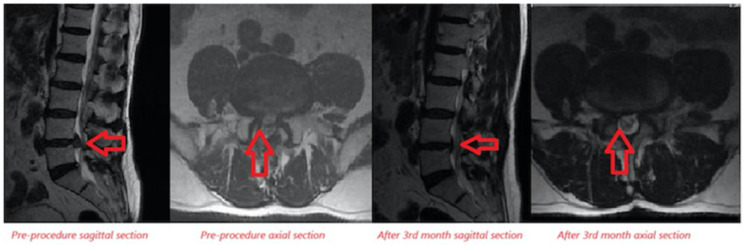
Examination of disc size reduction after ozone therapy in lumbar MRI sagittal and axial sections. MRI, magnetic resonance imaging.

Lumbar MRI examinations conducted at the 3^rd^ month follow-up of the patients were compared to pre-treatment MRI assessments in patients who underwent OT. A decrease in disc size was observed in 9 patients, while no significant change in disc size was noted in 14 patients, and an increase in disc size was determined in 2 patients (Figures 3, 4, and Table 4).

**Figure 5:**
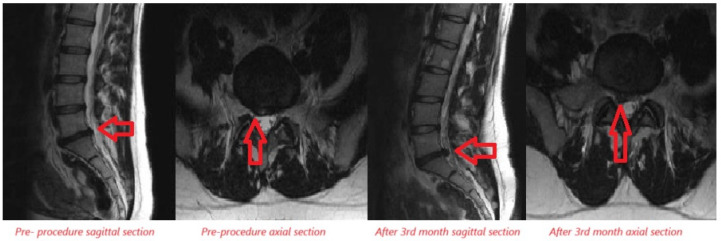
Examination of disc size increase after ozone + PRP combine therapy in lumbar MRI sagittal and axial sections. MRI, magnetic resonance imaging; PRP, platelet-rich plasma.

**Figure 6:**
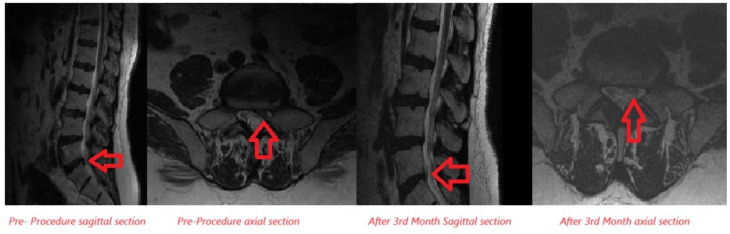
Examination of disc size reduction after ozone + PRP combine therapy in lumbar MRI sagittal and axial sections. MRI, magnetic resonance imaging; PRP, platelet-rich plasma.

Lumbar MRI examinations conducted at the 3^rd^ month follow-up of the patients were compared to pre-treatment MRI assessments in patients receiving ozone + PRP combined therapy. A decrease in disc size was noted in 5 of the patients who received OT, while no significant change in disc size was noted in 18 patients, and an increase in disc size was noted in 2 patients (Figures 5, 6 and Table 4).

Although the group receiving ozone combined with PRP exhibited a lower percentage of patients with reduced disc size compared to the ozone-only group, the more pronounced clinical improvements (VAS and ODI scores) indicate that disc size reduction alone may not fully account for the therapeutic benefits of the combination therapy.

### Outcome Assessment

**Figure 7:**
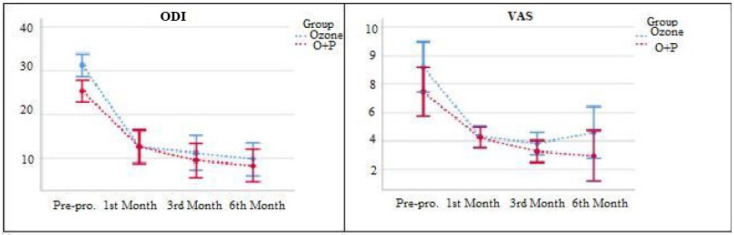
Comparison of ozone and ozone + PRP combine therapies. ODI, oswestry disability index; VAS, visual analog scale; PRP, platelet-rich plasma.

In the repeated measures ANOVA test, comparing OT and ozone + PRP therapy results in terms of VAS scores, F (1.49)=2.549, *P*=0.117 was obtained. As a result of comparing the results of OT and ozone + PRP in terms of ODI scores, it was found that F (1.49)=1.190, *P*=0.281. These results indicate no statistically significant difference in VAS and ODI values between the two treatment methods (*P *> 0.05), suggesting no superiority of one treatment method over the other (Figure 7).

### Safety and Adverse Events

Both treatment groups were well-tolerated, with no major adverse events reported. No cases of infection, nerve damage, or allergic reactions were observed. High doses of ozone can result in adverse effects such as disc tissue toxicity and systemic complications.^11, 14^ In our study, no cases of spondylodiscitis or other infections were observed; such complications have been documented in the literature.^15, 16^ Ensuring precise dosing and adherence to safety protocols is essential for mitigating these risks and enhancing the overall safety profile of OT. Additionally, 4 patients (2 from each group) experienced worsening of symptoms due to increased disc size and were referred for surgical intervention (Table 4).

## Discussion

The primary objective of this study was to evaluate and compare the clinical and radiological outcomes of intradiscal OT versus a combination of intradiscal ozone and PRP therapy in patients with IVDD. Our results indicated that both treatment methods resulted in significant improvements in pain and disability scores, and there is no difference in clinical benefit between ozone and the combination therapy.

### Clinical Outcomes

Our study revealed significant reductions in VAS and ODI scores for both the OT and ozone + PRP combined therapy groups at 1, 3, and 6 months post-treatment. Specifically, the VAS scores in the ozone group showed a gradual decrease, with the lowest scores recorded at the 3-month follow-up (Table 2). This aligns with the results of Muto et al.,^11^ who reported significant pain relief following intradiscal OT for lumbar disc herniation.^17^ Histopathological examination of intervertebral disc spaces in animal studies has shown increased regeneration in the group treated with OT compared to the degenerated control group.^18^

Numerous studies have revealed long-term improvements in patient-reported pain and movement function following intradiscal PRP injections.^19, 20, 21^ This suggests that the addition of PRP may enhance the overall efficacy of OT. This result is supported by recent studies, indicating that PRP has the potential to augment the effects of other treatments by enhancing tissue repair and mitigating inflammation.^22^ Notably, PRP is recognized for its ability to deliver growth factors and cytokines that can facilitate tissue regeneration and reduce inflammation, potentially contributing to its synergistic effects when combined with OT.

### Radiological Outcomes

Radiological evaluations conducted at the three-month follow-up indicated a reduction in disc size among 9 patients (36%) in the ozone treatment group and 5 patients (20%) in the ozone + PRP group (Table 4). These results align with the findings of Muto et al.,^11^ who documented a decrease in disc volume following OT. The observation that a higher proportion of patients in the ozone-only group experienced disc shrinkage, may reflect variability in individual treatment responses influenced by factors such as the severity of disc degeneration and the specific injection techniques employed.^23^ Notably, while both treatment modalities resulted in significant clinical improvements, the radiological changes did not consistently predict pain relief. This inconsistency mirrors the findings of Lehnert et al.,^24^ who reported no direct correlation between reductions in disc size and pain alleviation following OT. In contrast, Negro et al.^25^ found a significant association between disc shrinkage and reduced pain levels, suggesting that while disc volume reduction may contribute to pain relief, it is not the sole factor involved.

### Synergistic Effects of Ozone + PRP

Although our study did not reveal a statistically significant difference between the ozone and ozone + PRP groups, we posit that PRP exerts a synergistic effect, particularly within the combined treatment group. An experimental animal study demonstrated that intradiscal PRP treatment, administered following IVDD, resulted in the preservation of disc morphological characteristics and delayed degeneration, accompanied by reduction in the migration of inflammatory cytokines.^26, 27, 28^

### Limitations and Future Directions

The need for fluoroscopy and/or tomography guidance could limit the feasibility of this treatment in the common rehabilitation setting due to radiation risk.^29^ A limitation of our study is the relatively short follow-up duration, which restricts our ability to fully assess long-term outcomes and the sustainability of treatment effects. Future studies with extended follow-up periods and larger sample sizes are essential to validate these findings and to evaluate the long-term efficacy and safety of combined ozone and PRP therapy.

## Conclusion

In this study, we observed a significant reduction in pain scores following treatment in both the ozone and ozone + PRP groups, attributable to IVDD. This suggests that these treatment modalities may serve as viable alternatives to surgical intervention, particularly for patients without neurological deficits. The integration of diverse treatment approaches aims to facilitate the regeneration of disc tissue, alleviate associated pain, and enhance patients’ overall quality of life. There is a need to develop and experimentally evaluate new treatment modalities.

## Ethics

**Ethics Committee Approval:** Ethical approval was received from the University of Health Sciences Türkiye, Sultan 2. Abdülhamid Han Training and Research Hospital, GETAT Clinical Research Ethics Committee (approval no.: SBllSAH-GETAT 2023-048, date: 13.12.2023).

**Informed Consent:** Written informed consent was obtained from all patients’ legal representatives included in the study.

## Figures and Tables

**Table 1. Distributions of Variables:** 

**Group**	**Normality Tests**	**Kolmogorov-Smirnov**		**Shapiro-Wilk**	
		**Statistic**	**Sig.**	**Statistic**	**Sig.**
**Ozone**	Pre-Procedure VAS	0.408	**0.000**	0.378	**0.000**
	Pre-Procedure ODI	0.112	0.200*	0.956	0.343
	After 1^st^ month VAS	0.209	**0.006**	0.924	0.063
	After 1^st^ month ODI	0.208	**0.007**	0.870	**0.004**
	After 3^rd^ month VAS	0.223	**0.002**	0.893	**0.013**
	After 3^rd^ month ODI	0.190	**0.021**	0.838	**0.001**
	After 6^th^ month VAS	0.309	**0.000**	0.440	**0.000**
	After 6^th^ month ODI	0.184	**0.028**	0.840	**0.001**
**Ozone + PRP**	Pre-Procedure VAS	0.234	**0.001**	0.919	**0.043**
	Pre-Procedure ODI	0.111	0.200*	0.932	0.086
	After 1^st^ month VAS	0.241	**0.000**	0.926	0.063
	After 1^st^ month ODI	0.179	**0.032**	0.879	**0.005**
	After 3^rd^ month VAS	0.325	**0.000**	0.790	**0.000**
	After 3^rd^ month ODI	0.290	**0.000**	0.728	**0.000**
	After 6^th^ month VAS	0.338	**0.000**	0.720	**0.000**
	After 6^th^ month ODI	0.243	**0.000**	0.734	**0.000**

*SPSS Kolmogorov-Smirnov test *P *normality

Bold values: *P *< 0.200

VAS, visual analog scale; ODI, oswestry disability index; Sig., significant; PRP, platelet-rich plasma

**Table 2. Comparison of Patients Receiving Ozone Therapy in Terms of Periods:** 

**Ozone**	**Median (Min.-Max.)**	***P* value**	**Difference**
Pre-Procedure VAS	8 (5-38)	<0.001	**1>4 1>3 1>2**
After 1^st^ month VAS	4 (1-8)		
After 3^rd^ month VAS	3 (1-8)		
After 6^th^ month VAS	3 (1-33)		
Pre-Procedure ODI	31 (11-46)	<0.001	
After 1^st^ month ODI	10 (2-35)		
After 3^rd^ month ODI	8 (1-35)		
After 6^th^ month ODI	7 (1-35)		

1, pre-procedure; 2, after 1^st^ month; 3, after 3^rd^ month; 4, after 6^th^ month.

VAS, visual analog scale; ODI, oswestry disability index; Min.-Max., minimum-maximum

**Table 3. Comparison of Patients Receiving Ozone + PRP Combine Therapy in Terms of Periods:** 

**Ozone + PRP**	**Median (Min.-Max.)**	***P* value**	**Difference**
Pre-Procedure VAS	8 (5-10)	<0.001	**1>4 1>3 1>2**
After 1^st^ month VAS	4 (1-9)		
After 3^rd^ month VAS	3 (1-9)		
After 6^th^ month VAS	3 (1-9)		
Pre-Procedure ODI	25.5 (17-33)	<0.001	**2>41>41>31>2**
After 1^st^ month ODI	10.5 (1-36)		
After 3^rd^ month ODI	5.5 (2-36)		
After 6^th^ month ODI	3.5 (1-36)		

1, pre-procedure; 2, after 1^st^ month; 3, after 3^rd^ month; 4, after 6^th^ month.

VAS, visual analog scale; ODI, oswestry disability index; Min.-Max., minimum-maximum

**Table 4. Post-Treatment Evaluation of Disc Dimensions in Control Lumbar MRI:** 

**Lumbar MRI Disc Dimensions**	**Ozone Therapy (Number of patients)**	**Ozone + PRP Combine Therapy (Number of patients)**
Increase in disk size	2	2
No change in disk size	14	18
Reduction/decrease in disk size	9	5

MRI, magnetic resonance imaging; PRP, platelet-rich plasma
